# Analysis of influencing factor of coexisting prediabetes and prehypertension in adult residents of Jilin Province

**DOI:** 10.1186/s12902-018-0316-5

**Published:** 2018-11-26

**Authors:** Xiaowei Wang, Mingjie Wang, Shuangshuang Shao, Yang Zhang, Siyu Liu, Yue Gao, Yuhang Shen, Pinghui Sun

**Affiliations:** Epidemiology and Statistics, School of Public Health, Jilin University, Changchun, 130021 China

**Keywords:** Pre-diabetes, Pre-hypertension, Influencing factors

## Abstract

**Background:**

To explore the risk factors of coexisting prediabetes and prehypertension, to provide theoretical basis for early intervention.

**Methods:**

A multi-stage stratified random cluster sampling method was used to randomly select adult residents from Jilin Province in 2013 for questionnaire surveys, physical examinations, and laboratory tests.

**Results:**

The prevalence of coexisting prediabetes and prehypertension in Jilin Province was 11.3%. The binary Logistic regression results showed that age, sex, education, triglyceride (TG), BMI, waist circumference and alcohol consumption were the effects of factor coexisting prediabetes and prehypertension.

**Conclusion:**

It is important to pay attention to the early stage of hypertension and diabetes, control the transition from prehypertension and prediabetes to hypertension and diabetes, and improve the health of residents.

## Background

Studies have found that a high proportion of prehypertension and prediabetes patients progress to hypertension and diabetes [1–6]. In light of the world’s population growth and aging, hypertension is a global public health issue. Many studies have shown associations between pre-hypertension and a higher risk of the future development of hypertension and cardiovascular disease in general populations. However, pre-hypertension per se is not a disease with an immediate high risk, and the clinical value of the identification of pre-hypertension is the potential detection of the early stage of the risk of hypertension and/or cardiovascular disease over an individual’s lifespan. Data from 1,129,098 participants were derived from 20 prospective cohort studies. Prehypertension significantly increased the risk of CVD, CHD, and stroke mortality (RR 1.28, 95% CI 1.16–1.40; RR 1.12, 95% CI 1.02–1.23; and RR 1.41, 95% CI 1.28–1.56, respectively) Prediabetes (intermediate hyperglycaemia) is a high-risk state for diabetes that is defined by glycaemic variables that are higher than normal, but lower than diabetes thresholds. 5–10% of people per year with prediabetes will progress to diabetes, with the same proportion converting back to normoglycaemia. Prevalence of prediabetes is increasing worldwide and experts have projected that more than 470 million people will have prediabetes by 2030. And prediabetes, diabetes and prehypertension, hypertension will affect each other and interact with each other, resulting in a multiplier effect on cardiovascular disease [7–11].The research of Jie Wu [12] et al. showed that the prevalence of coexisting prediabetes and prehypertension in adult in north and northeastern China was 11.1%, and Luo Xiaojia [13] et al. showed that the prevalence of coexisting prediabetes and prehypertension in the middle-aged and elderly population in Chengdu was 12.5%, and the standardized prevalence was 11.5%. Prehypertension and prediabetes are good opportunities to control progression to hypertension and diabetes. Currently, coexisting prediabetes and prehypertension studies are relatively rare. This study aims to identify coexisting prediabetes and prehypertension related risk factors and provide theoretical basis for early intervention.

## Objects and methods

### Subjects

A multi-stage stratified random cluster sampling method was used to randomly select residents of 18 years of age or older who lived in the area for more than 6 months in Jilin Province in 2013 to conduct questionnaire surveys, physical examinations, and laboratory tests. A total of 4689 cases were recovered, of which 4143 were valid.

## Research methods

### Questionnaire survey

According to the 2010 China chronic disease and its risk factor monitoring questionnaire [14], after the increase or decrease of some items, a professionally trained investigator adopted a unified questionnaire and fills in a questionnaire to conduct a questionnaire survey. The main contents included: general demographic characteristics (age, gender, ethnicity, education, marital status, occupation, place of birth), eating habits (vegetables, meat intake, smoking, drinking) and biochemical indicators about diabetes or hypertension fasting plasma glucose (FPG), 2-h oral glucose tolerance test (2hOGTT), systolic, diastolic, total cholesterol (TC), triglyceride (TG), high-density lipoprotein cholesterol (HDL-L), low density lipoprotein cholesterol (LDL-L)).

#### Physical examination

The physical examination used a uniform calibration instrument. Each body measurement was performed by two surveyors. Height and weight were measured using height and weight scales. The subjects were asked to stand barefoot on the instrument, visually in front, back straight, weight accurate to 0.1 kg, height accurate to 0.01 m, using weight/height ^2^ (kg/m^2^)to calculate BMI, BMI < 18.5 for low body weight, BMI between 18.5–24 for the normal, BMI ≥ 24 for overweight; The waist circumference was measured with a tape measure, and the subject was required to stand naturally, with both feet separated and shoulder width apart. The tape measure was placed on the midpoint of the lower rib and the ridge line of the subject (usually the narrowest part of the waist). Horizontally around the abdomen, measuring the length of the waist circumference at the end of normal exhalation, accurate to 0.1 cm, abdominal obesity is defined as male waist circumference ≥ 85 cm, female waist circumference ≥ 80 cm;The blood pressure was measured with an Omron HEM-7071 electronic sphygmomanometer. The subjects were required to have no strenuous exercise within 1 h before measurement, no smoking, drinking, and drinking coffee. Measured in a quiet and relaxed state, each measured 3 times, requiring two measurement intervals at least 2 min.

#### Laboratory tests

Taking 7 ml of venous blood from an empty stomach for more than 10 h in the morning and sending to a laboratory for FPG(5.63 ± 1.149), TC(4.78 ± 0.980), TG(1.67 ± 1.395), HDL-L(1.30 ± 0.378), LDL-L(2.99 ± 0.857) and other indicators. After the first venous blood was drawn, 72 g of glucose was taken orally for 2 h before being drawn again to the laboratory for blood glucose testing. The abnormal values were defined as: TC ≥ 6.2 mmol/L (hypercholesterolemia), TG ≥ 2.3 mmol/L (hypertriglyceridemia), and HDL-L < 1.0 mmol/L (high-density lipoproteinemia) LDL-L ≥ 4.1 mmol/L (low-density lipoproteinemia) [15].

### Diagnostic criteria

#### Prediabetes

Prediabetes, impaired glucose regulation (IGR) includes impaired fasting glucose (IFG) and impaired glucose tolerance (IGT). According to the 1999 Diabetes and IGT/IFG Blood Glucose Diagnostic Criteria of the WHO [16], prediabetes was defined as fasting blood glucose at 6.1–7.0 mmol/L and 2-h oral glucose tolerance test less than 7.8 mmol/L, or fasting blood glucose less than 7.0 mmol/L and the 2-h oral glucose tolerance test was between 7.81 and 11.1 mmol/L (see Table 1).

**Table 1:** Diagnostic criteria for Blood glucose

Glucose metabolism states	Classification standard(mmol/L)
Normal blood glucose	FPG<6.1&2hPG<7.8
Prediabetes	IFG(6.1 ≤ FPG<7.0 & 2hPG<7.8)/ IGT(FPG<7.0 & 7.8 ≤ 2hPG<11.1)
Diabetes	FPG ≥ 7.0/2hPG ≥ 11.1

#### Prehypertension

According to the 2010 Hypertension Prevention Guidelines [17], prehypertension refers to systolic blood pressure between 120 and 140 mmHg, with or without diastolic blood pressure between 80 and 90 mmHg (see Table 2).

**Table 2:** Diagnostic criteria for blood pressure

Blood pressure level	Classification standard**(**mmHg**)**
Normal blood pressure	SBP<120&DBP<80
Prehypertension	120 ≤ SBP<140/80 ≤ DBP<90
Hypertension	SBP ≥ 140/DBP ≥ 90

### Statistical analysis

Using EpiData3.1 to establish a database and enter data, and using software SPSS21.0 for statistical analysis. In the single factor analysis, the qualitative data was tested with chi-square test, and the partition of Chi-square for multiple comparison. The quantitative data was tested with the rank sum test. Because the sample size was larger, to obtain more accurate results, the rank transform analysis was used for multiple comparison. Multivariate analysis was performed using binary Logistic regression analysis. Except for the partition of Chi-square, all analysis significance levels were 0.05.

## Results

### Classification of hypertension and diabetes in adults

Of the 4143 adults in this study, 470 cases coexisting prediabetes and prehypertension, accounting for 11.3% of the study population; 933 cases had both normotension and normoglycemia, accounting for 22.5% of the study population; 177 cases had only prediabetes, accounting for 4.3% of the study population; 1418 cases had only prehypertension, accounting for 34.2% of the study population. The results are shown in Table 3.

**Table 3:** Classification of Hypertension and Diabetes in Adults (*N* = 4143)

Type of metabolism	Normal blood pressure	Prehypertension	Hypertension	Total
Normal blood glucose	933(22.5)	1418(34.2)	520(12.6)	2871(69.30)
Prediabetes	177(4.3)	470(11.3)	250(6.0)	897(21.65)
Diabetes	40(1.0)	136(3.3)	199(4.8)	375(9.05)
Total	1150(27.76)	2024(48.85)	969(23.39)	4143(100)

### The cardiovascular risk factors of coexisting prediabetes and prehypertension

There were differences in age, gender, place of residence, education level, marital status, occupation, smoking, fruit juice consumption, total cholesterol (TC), triglyceride (TG), high-density lipoprotein cholesterol (HDL-L), low-density lipoprotein cholesterol (LDL-L), BMI, waist circumference and other factors by comparing the normal group, the group coexisting prediabetes and prehypertension, the only prediabetic group, and the only prehypertension group. The results are shown in Table 4.

**Table 4:** Normal VS Prediabetes and prehypertension VS only prediabetes VS only prehypertension (*N* = 2998)

variables	Normal	Prediabetes and prehypertension	Only prediabetes	Only prehypertension	Χ^2^	*P*-value
Age ^abc^						
18–24	79(8.5)	12(2.6)	6(3.4)	52(3.7)	181.253	0.000
25–34	198(21.2)	47(10.0)	24(13.6)	159(11.2)		
35–44	263(28.2)	95(20.2)	41(23.2)	316(22.3)		
45–54	227(24.3)	126(26.8)	63(35.6)	414(29.2)		
55–64	116(12.4)	116(24.7)	29(16.4)	333(23.5)		
65-	50(5.4)	74(15.7)	14(7.9)	144(10.2)		
Gender ^ab^						
Men	240(25.7)	227(48.3)	50(28.2)	626(44.1)	110.562	0.000
Women	693(74.3)	243(51.7)	127(71.8)	792(55.9)		
Place of residence						
Urban	563(60.3)	258(54.9)	113(63.8)	741(52.3)	19.824	0.000
Rural	370(39.7)	212(45.1)	64(36.2)	677(47.7)		
Education level ^a^						
Primary or below	185(19.7)	156(33.2)	39(22.0)	464(32.7)	111.207	0.000
Junior middle school	305(32.7)	164(34.9)	64(36.2)	488(34.4)		
Senior high school	225(24.1)	96(20.4)	51(28.8)	311(21.9)		
College or higher	219(23.5)	54(11.5)	23(13.0)	155(10.9)		
Marital status ^a^						
Single	82(8.8)	20(4.3)	10(5.6)	85(6.0)	22.382	0.001
Married/cohabitation	778(83.4)	387(82.3)	148(83.6)	1177(83.0)		
Separation/divorce/widowhood	73(7.8)	63(13.4)	19(10.7)	156(11.1)		
Occupation ^a^						
Physical work ^1^	523(56.1)	269(57.2)	88(49.7)	910(64.2)	64.671	0.000
Mental work ^2^	255(27.3)	86(18.3)	32(18.1)	247(17.4)		
Retirement or other	155(16.6)	115(24.5)	57(32.2)	261(18.4)		
Smoking ^a^						
Yes	177(19.0)	119(25.3)	35(19.8)	388(27.4)	23.919	0.000
No	756(81.0)	351(74.7)	142(80.2)	1030(72.6)		
Drinking						
Yes	318(34.1)	186(39.6)	72(40.7)	553(39.0)	7.586	0.055
No	615(65.9)	284(60.4)	105(59.3)	865(61.0)		
TC ^a^						
Normal	896(96.0)	423(90.0)	166(93.8)	1310(92.4)	21.086	0.000
Abnormal	37(4.0)	47(10.0)	11(6.2)	108(7.6)		
TG ^ac^						
Normal	870(93.2)	352(74.9)	148(83.6)	1221(86.1)	91.595	0.000
Abnormal	63(56.8)	118(25.1)	29(16.4)	197(13.9)		
HDL-L ^ac^						
Normal	807(86.5)	344(73.2)	141(79.7)	1159(81.7)	37.681	0.000
Abnormal	126(13.5)	126(26.8)	36(20.3)	259(18.3)		
LDL-L ^a^						
Normal	880(94.3)	413(87.9)	162(91.5)	1288(90.8)	18.291	0.000
Abnormal	53(5.7)	57(12.1)	15(8.5)	130(9.2)		
BMI^abc^						
Normal(18.5–24)	570(61.2)	173(37.0)	92(52.0)	621(43.9)	143.294	0.000
Below normal(<18.5)	53(5.7)	7(1.5)	4(2.3)	36(2.5)		
Above normal(≥24)	309(33.2)	288(61.5)	81(45.8)	757(53.5)		
Waist circumference ^ab^						
Normal	844(90.5)	322(68.5)	157(88.7)	1054(74.3)	134.930	0.000
Above normal	89(9.5)	148(31.5)	20(11.3)	621(20.7)		
Carbonated beverage intake						
Never	645(69.1)	351(74.7)	128(72.3)	1055(74.4)	9.984	0.125
<250 ml	246(26.4)	103(21.9)	44(24.9)	317(22.4)		
≥ 250 ml	42(4.5)	16(3.4)	5(2.8)	46(3.2)		
Juice intake ^a^						
Never	631(67.6)	373(79.4)	127(71.8)	1079(76.1)	38.747	0.000
<250 ml	258(27.7)	89(18.9)	46(26.0)	311(21.9)		
≥ 250 ml	44(4.7)	8(1.7)	4(2.3)	28(2.0)		

^1^ Agriculture, forestry, animal Husbandry, production, and business services staff

^2^ National government agencies, Party organizations, enterprises, institutions, professional and technical personnel, students

^a^ Comparison of the two sides by the partition of Chi-square method: the group coexisting prediabetes and prehypertension was different from the normal group. *P*<0.00833

^b^ Comparison of the two sides by the partition of Chi-square method: the group coexisting prediabetes and prehypertension was different from the only prediabetes group. *P*<0.00833

^c^ Comparison of the two sides by the partition of Chi-square method: the group coexisting prediabetes and prehypertension was different from the only prehypertension group. *P*<0.00833

### Logistic regression analysis of coexisting prediabetes and prehypertension

The 17 variables, such as age, gender, nation, place of residence, education level, marital status, occupation, smoking, drinking, alcohol consumption, fruit juice intake, total cholesterol (TC), triglyceride (TG), high-density lipoprotein cholesterol (HDL-L), low density lipoprotein cholesterol (LDL-L), BMI, waist circumference and pork intake were included in the binary logistic regression model, and the significance level was set at 0.05.

### Comparison of prediabetes with prehypertension group and normal group

The binary logistic regression analysis was performed using prediabetes with prehypertension group(Y = 1)and normal group(Y = 0)as the dependent variable. The results showed that variables such as age, gender, education level, triglyceride (TG), BMI, and waist circumference were significant. The results are shown in Table 5.

**Table 5:** Logistic regression analysis of prediabetes with prehypertension group and normal group

Variables	Levels	P	OR(95%C.I. for OR)
Constant		0.000	0.196
Age_(control = 18–24)_	25–34	0.279	1.515(0.714–3.216)
	35–44	0.031	2.202(1.075–4.510)
	45–54	0.003	2.925(1.434–5.694)
	55–64	0.000	4.991(2.411–10.331
	65-	0.000	7.179(3.283–15.700)
Gender_(control = men)_	Women	0.000	0.525(0.366–0.751)
Education level_(control = Primary or below)_	Junior middle school	0.084	0.750(0.541–1.040)
	Senior high school	0.001	0.521(0.360–0.753)
	College or higher	0.000	0.406(0.263–0.624)
TG_(control = normal)_	Abnormal	0.000	3.292(2.281–4.751)
BMI_(control = 18.5–24)_	<18.5	0.035	0.390(0.163–0.936)
	≥24	0.000	2.688(2.037–3.545)
WC_(control = normal)_	Above normal	0.020	1.705(1.090–2.667)

### Comparison of prediabetes with prehypertension group and only prediabetes group

The binary logistic regression analysis was performed using prediabetes with prehypertension group(Y = 1)and only prediabetes group(Y = 0)as the dependent variable. The results showed that variables such as age, education, drinking, BMI, waist circumference, and pork intake were significant. The results are shown in Table 6.

**Table 6:** Logistic regression analysis of prediabetes with prehypertension group and only prediabetes group

Variables	Levels	P	OR**(**95%C.I.for OR**)**
Constant		0.000	5.501
Gender_**(**control = men**)**_	Women	0.010	0.477 (0.272–0.835)
Education level_**(**control = primary or below**)**_	Junior middle school	0.034	0.598(0.372–0.962)
	Senior high school	0.013	0.514 (0.304–0.868)
	College or higher	0.017	0.452 (0.236–0.866)
Drinking_**(**control = no**)**_	Yes	0.037	0.644 (0.426–0.973)
BMI_**(**control = 18.5–24**)**_	<18.5	0.768	0.824 (0.229–2.968)
	≥24	0.001	1.960 (1.308–2.938)
WC_**(**control = normal**)**_	Above normal	0.019	2.331 (1.152–4.713)
Pork intake		0.007	0.996 (0.993–0.999)

### Comparison of prediabetes with prehypertension group and only prehypertention group

The binary logistic regression analysis was performed using prediabetes with prehypertension group(Y = 1)and only prehypertension group(Y = 0)as the dependent variable. The results showed that variables such as age and triglyceride (TG) were significant. The results are shown in Table 7.

**Table 7:** Logistic regression analysis of prediabetes with prehypertension group and only prehypertension group

Variables	Levels	P	OR**(**95%C.I.for OR**)**
Constant		0.000	0.206
Age_(control = 18–24)_	25–34	0.599	1.211 (0.594–2.468)
	35–44	0.497	1.262(0.644–2.474)
	45–54	0.493	1.261 (0.650–2.449)
	55–64	0.272	1.454 (0.746–2.833)
	65-	0.020	2.282 (1.414–4.562)
TG_(control = normal)_	Abnormal	0.000	2.138 (1.648–2.772)

## Discussion

The survey results showed that the prevalence of prediebetes with prehypertension in Jilin residents was 11.3%, which was close to the results of Jie Wu et al. [12] who surveyed adults in North and Northeast China (11.1%) and Xiaojia Luo et.al [13]. who surveyed the middle-aged and elderly people in Chengdu(11.5%). It showed that the prevalence of prehypertension with prediabetes in Jilin residents was at a general level. However, studies have shown that the proportion of prehypertension to high blood pressure was 32.8% every 2 years [1], 38.0% every 3 years [3], the proportion of prediabetes to diabetes was 18.4% every 3 years [4]. Therefore, the interaction between pre-diabetes and diabetes and prehypertension and hypertension is still not to be underestimated, and the product effect of cardiovascular disease damage still needs attention.

Univariate analysis of pre-diabetes with pre-hypertension and normal subjects showed statistically significant differences in gender, age, education, marital status, occupational type, smoking and hyperlipemia、TC、TG、HDL-L、LDL-L、BMI、waist circumference and juice intake among the two groups. Logistic regression analysis of pre-diabetes with prehypertension group and normal group showed that high age group, hypertriglyceridemia, excessive BMI, and abdominal obesity were risk factors for pre-diabetes with prehypertension. Female gender and high education level are protective factors for pre-diabetes complicated with pre-hypertension. In the high-age group, especially in the group of 65 years and older, compared with normal blood pressure and normal blood glucose, the probability of having pre-diabetes with prehypertension is 7.179 times that of the 18–24 year old group. Old age chronic diseases will place a very heavy burden on families and society. It is suggested that we should pay close attention to blood pressure and blood glucose changes, regular physical examination, and early treatment, so as not to cause serious complications after disease progression. Most people with lower education levels have lower quality of life and less emphasis on health, so they are more likely to have pre-diabetes and pre-hypertension. The result may be due to the higher quality of life of people with higher education levels and the higher degree of emphasis on health, and thus the relatively lower risk of illness, suggesting that we should increase the popularity of health knowledge, as far as possible, taking into account all the people; Hypertriglyceridemia wass 3.292 times more likely to have prediabetes with prehypertension than normal blood triglyceride levels. The excessive BMI was 2.688 times more likely to have prediabetes with prehypertension than those with normal BMI. Abdominal obesity was 1.75 times more likely to have prediabetes with prehypertension than is normal.The results shown that hypertriglyceridemia, overweight, and abdominal obesity were all risk factors for prediabetes and prehypertension, which were the same as those of Xiaojia Luo [13].

Univariate analysis of pre-diabetes with prehypertension group and pre-diabetes group showed statistically significant differences in age, gender, BMI, and waist circumference between the two groups. Logistic regression analysis of the prediabetes with prehypertension group and only prediabetes group showed: Compared with the only prediabetes group, men, drinking, lower education, excessive BMI, abdominal obesity, and pork intake were positively associated with the prevalence of prediabetes with prehypertension. A large number of studies have shown that drinking was an important factor leading to prehypertension [18, 19], and it was also an important factor leading to prediabetes [20, 21], while prediabetes and prehypertension promote each other, and therefore drinkers are more prone to have prediabetes with prehypertension. Male drinkers are far higher than females, and the low level of education in health care is relatively weak. Excessive BMI and abdominal obesity both increase the risk of prediabetes with prehypertension, suggesting that peaple need to keep a good diet, moderate drinking, and have good habits. Since the OR value of pork intake was close to 1, it did not have much practical significance and therefore will not be discussed.

Univariate analysis of pre-diabetes with prehypertension group and pre-hypertension group showed that there was a statistically significant difference in age, TG, and HDL-L between the two groups. Logistic regression analysis of the prediabetes with prehypertension group and only prehypertension group showed: Compared with the only prehypertensive group, the higher age and hypertriglyceridemia was positively correlated with the prevalence of prehypertension with prediabetes. The risk of prediabetes with prehypertension in the 55–64-year-old group was 1.454 times higher than that in the 18–24-year-old group, and the risk of prediabetes with prehypertension in the 65-year-old group was 2.282 times higher than that in the 18–24 year-old group. Suggesting that we should pay attention to the health of middle-aged and elderly people. The risk of prediabetes with prehypertension in hypertriglyceridemia is 2.138 times than that in normal triglycerides. Studies have shown that hypertriglyceridemia is a risk factor for pre-diabetes [4], consistent with the results of this study. Evidence-based medicine showed that almost all diabetic patients went through the prediabetic stage, and about 1/3 of the prediabetic patients progressed to diabetes every 5 to 10 years [22], but the disease was highly reversible at an early stage, so care should be taken at the early stage of the disease to prevent the disease from developing to an uncontrollable level.

Some limitations of our study should be noted. First, respondents’ smoking status and drinking status were based on self-report, which may be subject to reporting bias. Second, some of the variables were not quantifiable, and thus more details could not be provided.

## Conclusions

In 2016, the prevalence of pre-diabetes with prehypertension in Jilin Province was at a general level. Pre-diabetes with pre-hypertension group compared with normal group, high age group, abnormal TG, high BMI and abdominal obesity were important risk factors; Pre-diabetes with prehypertension group compared with only pre-diabetes group, high BMI and abdominal obesity were important risk factors; Pre-diabetes with prehypertension group compared with only prehypertension group, high age group and abnormal TG were important risk factors. It is important to pay attention to the early stage of hypertension and diabetes, control the transition from prehypertension and prediabetes to hypertension and diabetes, and improve the health of residents.
